# Antioxidant enzymes in chickpea colonized by *Piriformospora indica* participate in defense against the pathogen *Botrytis cinerea*

**DOI:** 10.1038/s41598-017-12944-w

**Published:** 2017-10-19

**Authors:** Om Prakash Narayan, Nidhi Verma, Alok Kumar Singh, Ralf Oelmüller, Manoj Kumar, Durga Prasad, Rupam Kapoor, Meenakshi Dua, Atul Kumar Johri

**Affiliations:** 1 School of Life Sciences Jawaharlal Nehru University, New Meharuli Road, New Delhi, 110067 India; 2 School of Environmental Sciences, Jawaharlal Nehru University, New Meharuli Road, New Delhi, 110067 India; 3 Institute of Plant Physiology, Friedrich-Schiller-University Jena, Dornburger Str. 159, 07743 Jena, Germany; 4 Department of Botany, University of Delhi, Delhi, 110007 India

## Abstract

*Piriformospora indica*, a root endophytic fungus, promotes growth of the economically important chickpea plant (*Cicer arietinum* Linn.) and protects it against the pathogenic fungus *Botrytis cinerea*. Biomass and root development were found to be significantly improved in chickpea plants colonized with *P*. *indica* as compared to the plants grown without *P*. *indica* as well as from the plants infected with the *B*. *cinerea*. Our PCR analyses showed that gradual increase in the colonization of *P*. *indica* in the plants result in the inhibition of the colonization of *B*. *cinerea*. *P*. *indica* colonized plants showed increased antioxidant enzyme activities. Interestingly, there were pronounced decrease in the antioxidant enzyme activities in shoots infected with *B*. *cinerea* and colonized with *P*. *indica* in alternate and simultaneous mode as compared to plants infected with *B*. *cinerea* alone. We conclude that *P*. *indica* helps plants to overcome the disease load by enhancing antioxidant enzyme defense system. Our data suggest that, bio-protective action of *P*. *indica* might be mediated via systemic induction of antioxidant defense in the host plants.

## Introduction

Mycorrhizal associations are very important for a wide range of plants. They are broadly categorised into endomycorrhiza and ectomycorrhiza^1^. Arbuscular mycorrhiza (AM) association is found between a wide variety of plants e.g., in some bryophytes, gymnosperms, pteridophytes and angiosperms. Arbuscular Mycorrhizal Fungi (AMF) are obligate biotrophs and colonize root cells when they get a specific response from the plants. Through this association both partners fulfil their nutritional demands. In particular phosphate is given by the fungus to the host plant and in turn fungus gets reduced carbon from the plant^2^. The symbiotic interaction requires a balance between the plant defence and the nutritional requirements of both partners. Mycorrhized plants are also better protected against pathogens^3–5^.

It is generally believed that during early phases of beneficial plant/microbe interaction, plant activates defence responses and alters the antioxidant enzyme level, since it does not know yet whether it interacts with a friend or foe. The weak plant defence response includes defence gene activation and H_2_O_2_ production^6^. Stimulation of the anti-oxidative enzyme levels in the plant suggests that oxidative compounds are formed during the interaction with the fungus. Plants produce different types of antioxidative scavengers to cope with ROS^7^. The enzymatic ROS scavenging system includes sodium superoxide dismutases (SODs), glutathione peroxidases (GPXs), ascorbate peroxidases (APXs), catalases (CATs), glutathione-S-transferases (GSTs) and glutathione reductases (GRs).

*B*. *cinerea* is a necrotrophic pathogen that causes disease in many economically important cereal crops and medicinal plants all over the world. *B*. *cinerea* is the causative agent of Botrytis Grey Mould (BGM) disease in several plant species, including chickpea (*Cicer arietinum* Linn.). The asexual stage of *B*. *cinerea* is dominant in chickpea plant and the pathogen infects mainly the aerial part and the infection may lead to complete crop loss^8–10^.

Unlike AMF, *P*. *indica* is an axenically cultivable fungus that colonises a wide range of hosts without any host specificity^11^. *P*. *indica* colonizes plants belonging to bryophytes, pteridophytes, gymnosperms and a large number of monocots and dicots^12–14^. Colonization of *P*. *indica* in plants promotes biomass production, early flowering, seed production, nutrient uptake from the soil and provides tolerance against biotic and abiotic stresses^15–18^. It has been reported that *P*. *indica* protects barley from the adverse effects of the high salt concentration. *P*. *indica* associated plants showed a high level of antioxidant enzyme activities which protect plants from oxidative damage. *P*. *indica* inoculated plants showed an increase in SOD, GR, CAT and GST activities^19,20^ which help plants to overcome infections and stresses. They might also participate in promoting plant growth by keeping the ROS level below a critical threshold^21^. To know how *P*. *indica* help the infected chickpea plants to overcome disease load of *B*. *cinerea*, tripartite interaction of *P*. *indica*, *B*. *cinerea* and chickpea plants and the role of antioxidant enzymes have been investigated in this study.

## Results

### Role of *P*. *indica* in bioprotection against *B*. *cinerea*

**Figure 1:**
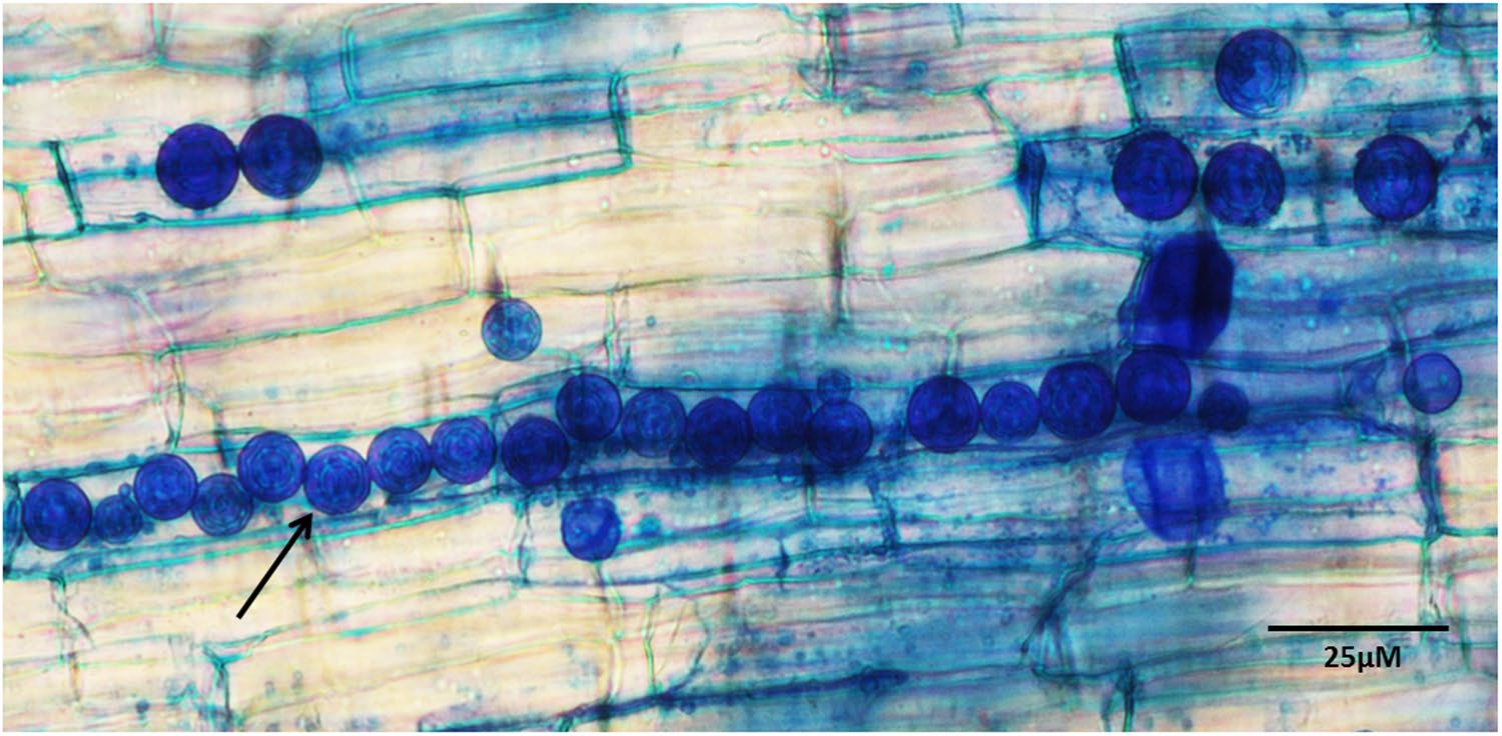
Histochemical analysis. Microscopic view of *P. indica* chlamydospores in cortical region of chickpea plant root stained with trypan blue after 15 days of colonization.

**Figure 2:**
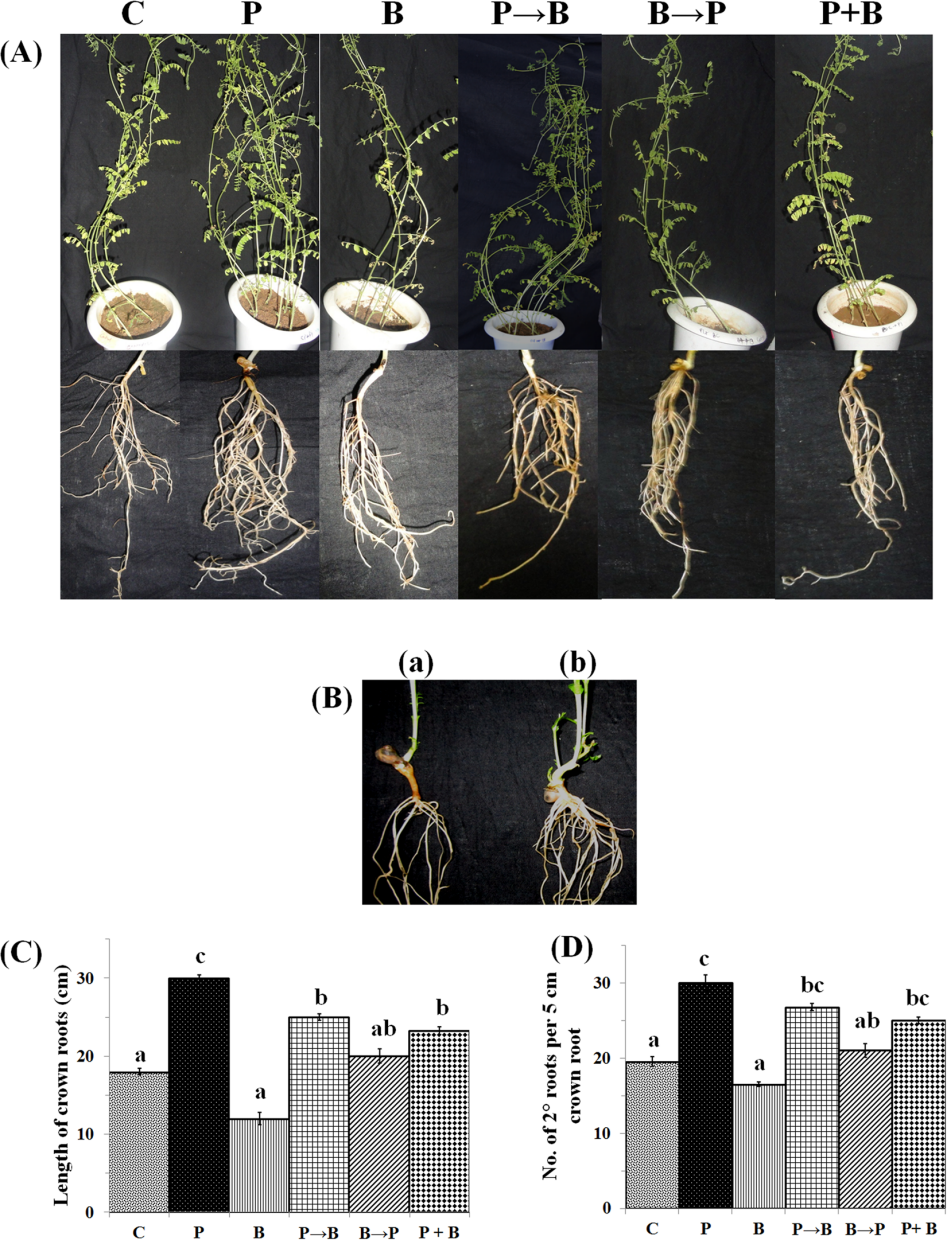
Morphological shoot/root growth pattern analysis in case of chickpea plant inoculated with *P. indica* and infected with *B. cinerea* in different experimental condition. (**A**) Plant shoot and root phenotype demonstrating the bioprotection role of *P. indica* against *B. cinerea*. Chickpea plants were grown for 30 days without any fungus were used as control (C); Chickpea plants inoculated with *P. indica* alone at day 0 and grown for 30 days (P); Chickpea plants infected with *B. cinerea* alone at day 0 and grown for 30 days (B); Chickpea plants first inoculated with *P. indica* at day 0 and at day 10 infected with *B. cinerea* and grown for 30 days (P→B); Chickpea plants first infected with *B. cinerea* at day 0 and at day 10 inoculated with *P. indica* and grown for 30 days (B→P); Chickpea plants inoculated/infected simultaneously with both fungi at day 0 and grown for 30 days (P+B). (**B**) Root growth pattern in control plant without colonization (a); and in a plant colonized with *P. indica* (b). (**C**) Change in length of crown roots determined under similar condition mentioned above (**D**) Number of secondary roots determined under similar condition mentioned above; Six pots, each having four plantlets per pot were used to determine the secondary root numbers (n = 24). Different letters indicate significant differences in length of crown roots and number of secondary roots at *P* ≤ 0.05 (Tukey’s test).

We have observed that *P*. *indica* spores germinate and enter into the chickpea roots within 5 to 7 days. Further we have found that colonization of the roots depends on the size of the inoculum and duration of co-cultivation. We have observed minimum 7–9% of colonization by the *P*. *indica* in the roots of chickpea plants in 7 days and maximum 65% colonization after 30 days of interaction (Table 1 **)**. We have observed intracellular densely packed pear shaped chlamydospores in the plant roots (Fig. 1). We observed distinct morphological alterations in case of roots as well as in shoots of plants inoculated with *P*. *indica* as compared to the plants without *P*. *indica* (control) (Fig. 2A). In case of colonized plants by *P*. *indica*, we have found an increase in the length of primary, seminal, crown and lateral roots. We have also observed increased number of seminal and crown roots in case of plants inoculated with *P*. *indica* as compared to the plants grown alone (Fig. 2B). During later stages fine secondary and tertiary roots formed interwoven network (Fig. 2A,B). In contrast, plants infected with *B*. *cinerea* showed poor root and shoot growth as compared to non-infected plants (Fig. 2A). Significantly, more number of secondary roots and increased crown root length was observed in case of plants colonized with the *P*. *indica* as compared to the plants infected with the *B*. *cinerea* as well as with that of plants not inoculated with either of the fungus (P < 0.05) (Fig. 2C,D). Also crown root length as well as secondary roots number were found to be significantly more in case of control plants (not inoculated with either of the fungus) as compared to the plants only infected with the *B*. *cinerea* (P < 0.05). We have observed increased length of crown roots and more number of secondary roots in case of plants first inoculated with *P*. *indica* and 10 days later infected with *B*. *cinerea* as compared to chickpea plants which were not colonized either with *B*. *cinerea* or *P*. *indica* and this difference was found to be significant (P < 0.05) (Fig. 2C,D). A similar trend was also observed when the plants were first infected with *B*. *cinerea* and at day 10 inoculated with *P*. *indica*, or when both fungi were applied simultaneously as compared to the plants infected with the *B*. *cinerea* only (P < 0.05) (Fig. 2C,D).

**Table 1:** Percent colonization of *P. indica* in chickpea roots.

**Time after inoculation (days)**	**% colonization**
**5**	**8** ± 4.2
**15**	**28** ± 1.5
**30**	**65** ± 1.5

Note: Plants were inoculated with *P. indica* culture (1 gm/pot) and roots were harvested at 5, 15, 30 days after inoculation. From one plant 10 roots were selected and chopped into 10 pieces. Percentage colonization was calculated by the presence of a chlamydospore in each piece.

**Figure 3:**
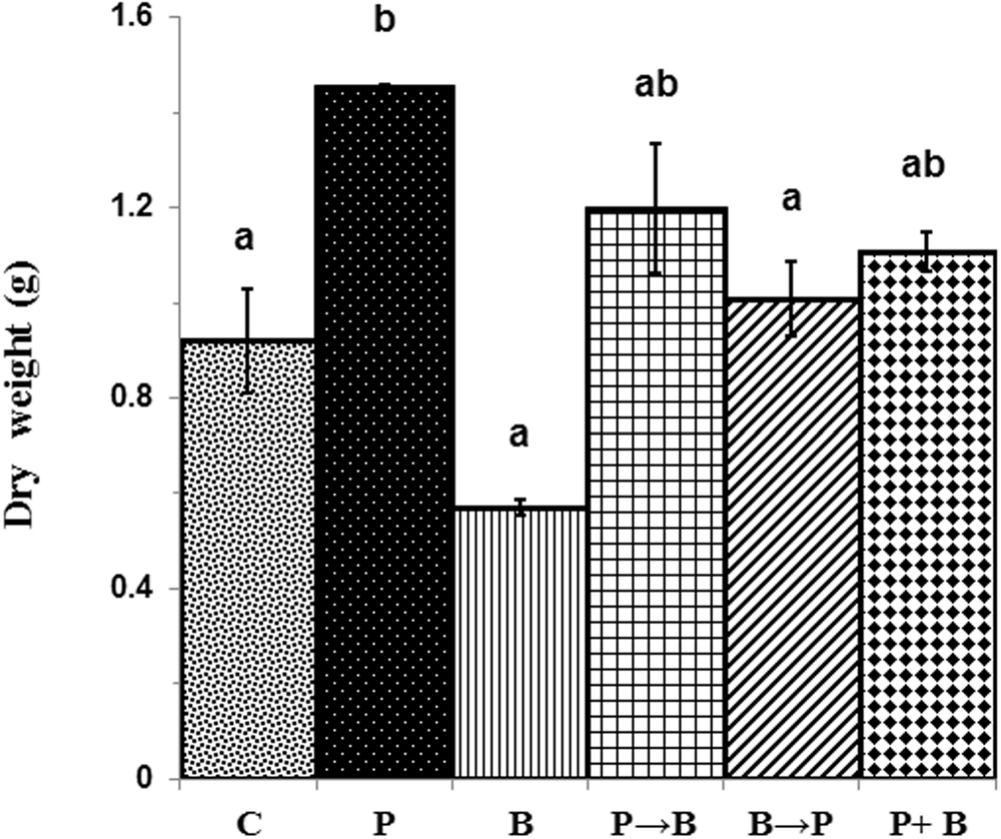
Impact of colonization of *P. indica* on biomass yield. Impact of simultaneous and alternate inoculation of *P. indica* and *B. cinerea* on biomass yield **(**dry weight) relative to plants exposed to *P. indica* or *B. cinerea* alone or not exposed to any fungus at 30 days. Different letters indicate significant differences in biomass yield at *P* ≤ 0.05 (Tukey’s test).

In case of dry weight, we found a significant increase, i.e. 1.6-fold, in 30-day-old chickpea plants colonized with *P*. *indica* as compared to the plants grown without fungus (P < 0.05) (Fig. 3). We observed a 0.22-fold decrease in the dry weight of 30-day-old chickpea plants infected with *B. cinerea* (P < 0.05) as compared with non-colonized plants (control). An improved biomass yield, i.e. a 1.8-fold increase in dry weight was observed in the plants first inoculated with *B. cinerea* and later inoculated with the *P. indica* as compared to the plants inoculated with *B. cinerea* only (Fig. 3) (P < 0.05). Further, we found, a 1.25-fold increase in dry weight in case of chickpea plants inoculated together with *P. indica* and *B. cinerea* at day 0 in comparison with chickpea plants grown without any fungus (Fig. 3 **)**. We have not observed any harmful effects of *B. cinerea* on the overall plant health in case of plants first inoculated with the *P. indica* and later infected with *B. cinerea* (Figs 2, 3).

**Figure 4:**
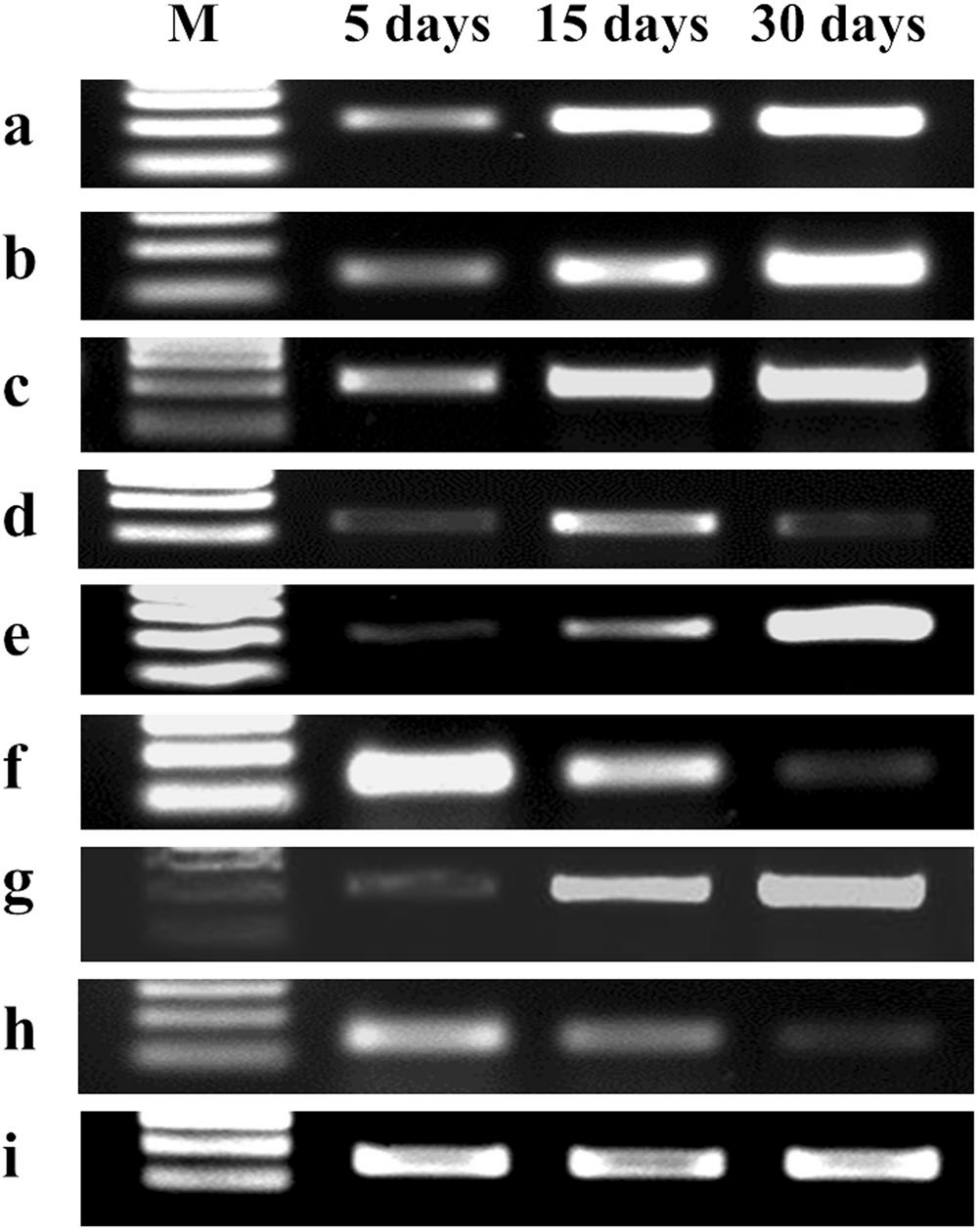
Interaction of *P. indica* and *B. cinerea* with chickpea plants. Amplification of DNA from chickpea roots after 5, 15 and 30 days of alternate inoculation of *P. indica* and *B*. *cinerea*. (**a**) Amplification of the *P. indica tef* gene from plants inoculated with *P. indica* alone. (**b**) Amplification of the *B. cinerea cpr1* gene from plants inoculated with *B. cinerea* alone. (**c**) Amplification of the *P. indica tef* gene from *P. indica* inoculated plants later infected with *B. cinerea*. (**d**) Amplification of the *B. cinerea cpr1* gene from *P. indica* inoculated plants later infected with *B. cinerea*. (**e**) Amplification of the *P. indica tef* gene from *B. cinerea* infected plants later inoculated with *P. indica*. (**f**) Amplification of the *B. cinerea cpr1* gene from *B. cinerea* infected plants later inoculated with *P. indica*. (**g**) Amplification of the *P. indica tef* gene from *P. indica* inoculated and *B. cinerea* infected plants simultaneously. (**h**) Amplification of the *B. cinerea cpr1* gene from *P. indica* inoculated and *B. cinerea* infected plants simultaneously. (**i**) Amplification of chickpea elongation factor 1-alpha (*EF1-α*) gene (control). M- 100 bp DNA ladder.

A gradual increase in the band intensity of the *tef* gene was observed in case of *P. indica-* inoculated plants at 5, 15 and 30 days (Fig. 4a). We have obtained similar results for *cpr1* gene in the case of plants inoculated with *B. cinerea* alone (Fig. 4b). In case of plants first inoculated with *P. indica* and later infected with the *B. cinerea*, we have observed a gradual increase in the band intensity of *tef* gene till 30 days, however in case of *cpr1* gene only till day 15 band intensity was slightly increased. No band of *cpr1* gene was observed at day 30 (Fig. 4c,d). Further we found an increase in the band intensity of *tef* gene till 30 days in case of plants first inoculated with *B. cinerea* and later inoculated with *P. indica* (Fig. 4e). Importantly, we observed a gradual decrease in the band intensity of the *cpr1* gene in the same samples and at the end of the 30 days a very faint band was observed (Fig. 4f).

In case of plants inoculated simultaneously with *P. indica* and *B. cinerea*, we have observed a gradual increase in the band intensity of *tef* gene till 30 days, but in case of *cpr1* a marked decrease in the band intensity was found and at the day 30, a very light band was observed (Fig. 4g,h).

### Antibiosis assay of *P. indica* and *B. cinerea*

**Figure 5:**
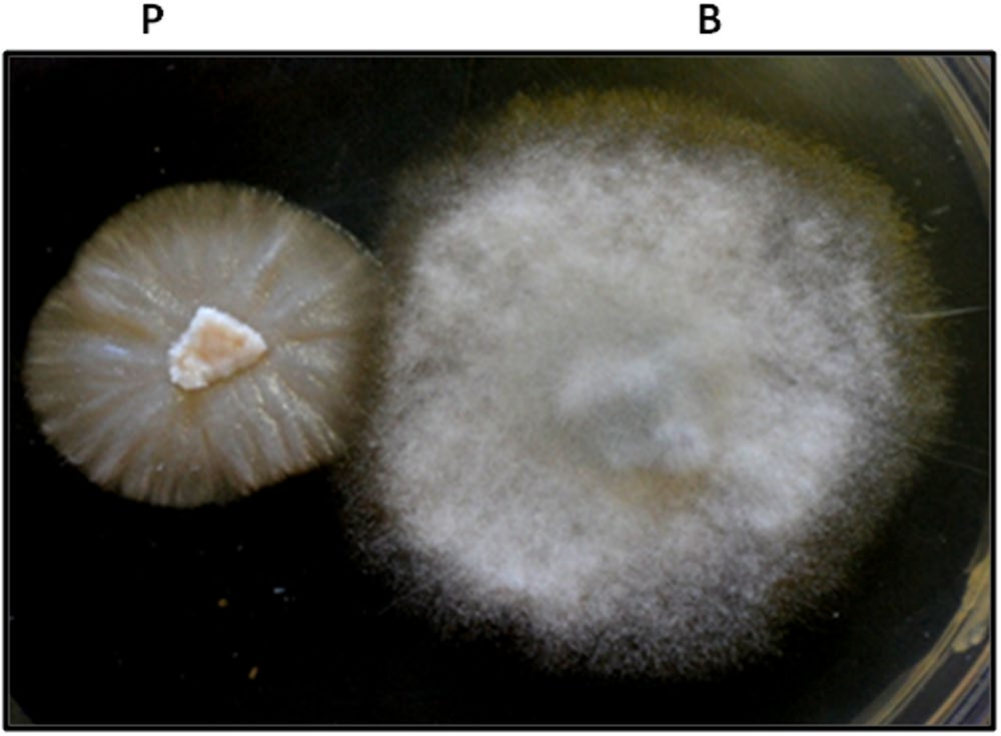
Antibiosis assay. Co-cultivation of *P. indica* (P) with *B. cinerea* (B) on KF –agar plate.

This assay was performed in order to check whether the increased resistance to *B. cinerea* is mediated by antibiotics/chemicals secreted by *P. indica* or not. We have not observed any growth inhibition of the two fungi when grown together which suggests that both fungi do not secret antibiotics or chemicals (Fig. 5).

### Antioxidant enzyme activities

**Figure 6:**
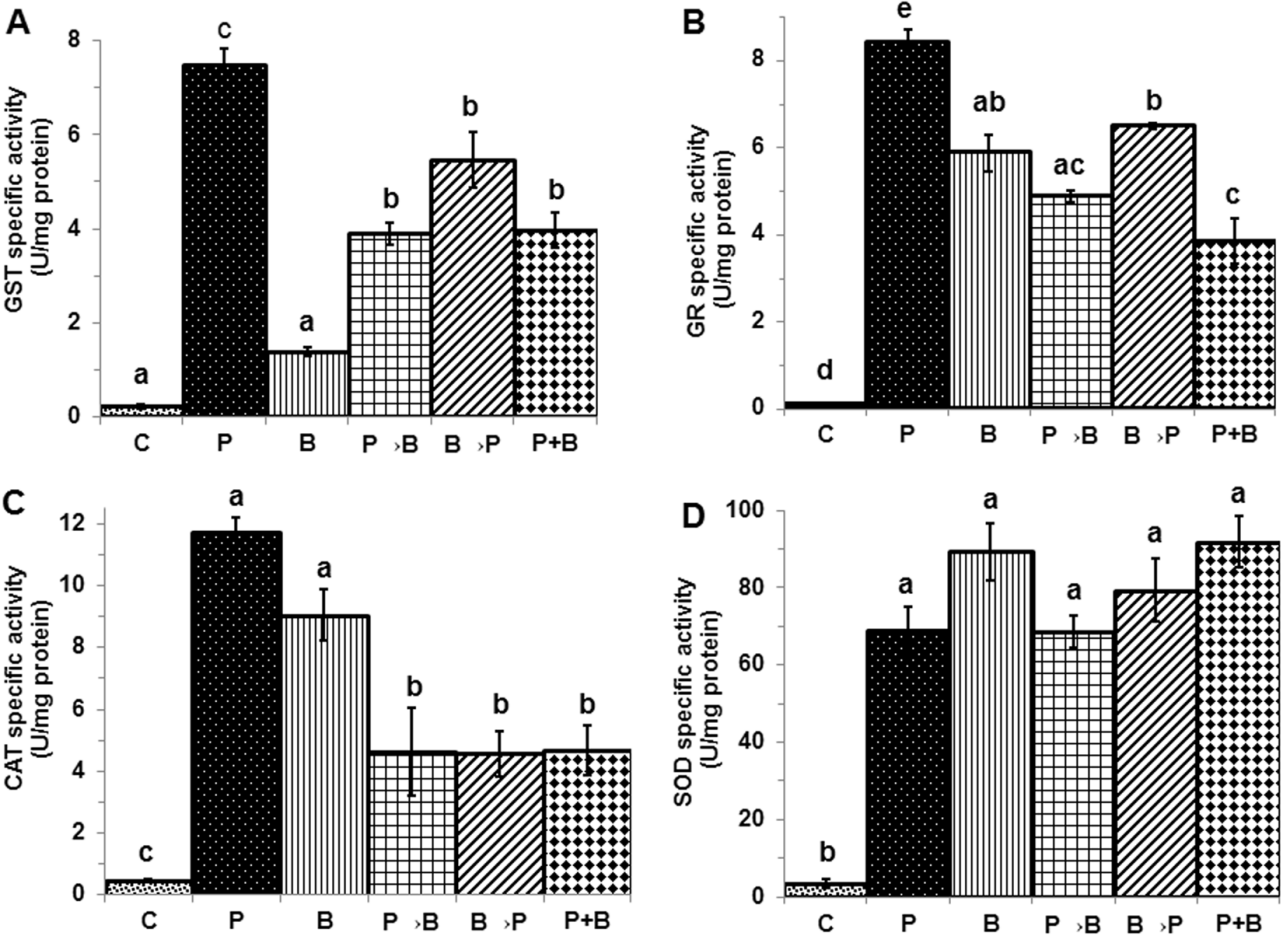
Antioxidant enzyme activities in chickpea roots. Activity of antioxidant enzymes in root of plants with alternate (P→B) and (B→P) and simultaneous (P+B) colonization/infection of *P. indica* (P) and *B. cinerea* (B). **(A)** GST, **(B)** GR, **(C)** CAT and **(D)** SOD activities. Specific activities compared with those of control plants not exposed to any fungus. Different letters indicate significant differences in different antioxidant enzymes at *P ≤ *0.05 (Tukey’s test).

In case of roots, antioxidant enzyme activities were found to be significantly increased i,e. 34, 71, 26 and 19 fold for GST, GR, CAT and SOD respectively in chickpea plants colonized with *P. indica* as compared to non-colonized plant (Fig. 6A–D) (P < 0.05). GST and GR activity were found to be significantly increased i,e. 6 and 50 folds respectively in case of plant infected with *B. cinerea* as compared to non-infected plant (P < 0.05), (Fig. 6A,B). Under similar conditions, in case of CAT and SOD no significant difference was found, though we observed an increase in the activity in case of both the enzymes (Fig. 6C,D). In case of simultaneous and alternate inoculation of *P. indica* and infection of *B. cinerea*, we found significant increase (P < 0.05) in the GST activity as compared to plant infected with *B. cinerea* only (Fig. 6A) while significantly (P < 0.05) decreased activity of CAT was observed (Fig. 6C). However, no significant differences were found in the activity of GR and SOD enzymes (Fig. 6B,D).

**Figure 7:**
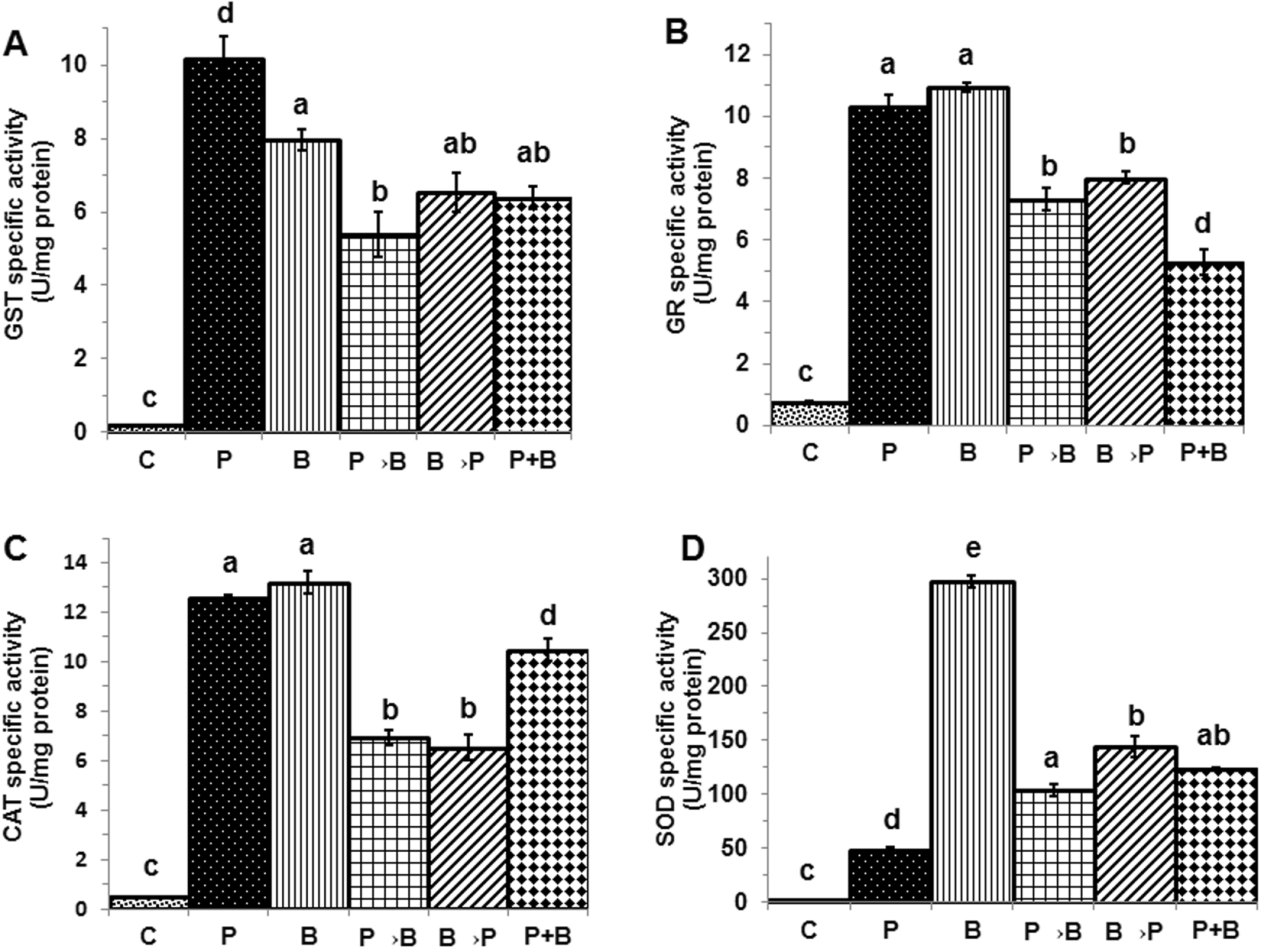
Antioxidant enzyme activities in chickpea shoots. Activity of antioxidant enzymes in shoot of plants with alternate (P→B and B→P) and simultaneous (P+B) colonization/infection of *P. indica* (P) and *B. cinerea* (B). **(A)** GST, **(B)** GR, **(C)** CAT and **(D)** SOD activities. Specific activities compared with those of control plants not exposed to any fungus. Different letters indicate significant differences in different antioxidant enzymes at *P* ≤ 0.05 (Tukey’s test).

In case of shoot, the antioxidant enzyme activities were found to be significantly increased i,e. 49, 13, 27 and 18 fold for GST, GR, CAT and SOD respectively in chickpea plants colonized with *P*. *indica* as compared to non-colonized plant (P < 0.05) (Fig. 7A–D). In case of simultaneous and alternate inoculation and infection of *P*. *indica* and *B*. *cinerea*, a decreased activity of GST, GR, CAT and SOD were found as compared to plant infected with *B*. *cinerea*, however, significant decreased activity was found in case of plants inoculated first with the *P*. *indica* and later infected with the *B*. *cinerea* as compared to the plants infected with the *B*. *cinerea* only (P < 0.05) (Fig. 7A–D **)**. In case of plants, infected with *B*. *cinerea* only, SOD activity was found to be significantly increased i,e. 110 fold as compared to plants not infected with *B*. *cinerea* (P < 0.05) (Fig. 7D).

### Role of *P*. *indica* in disease resistance

**Figure 8:**
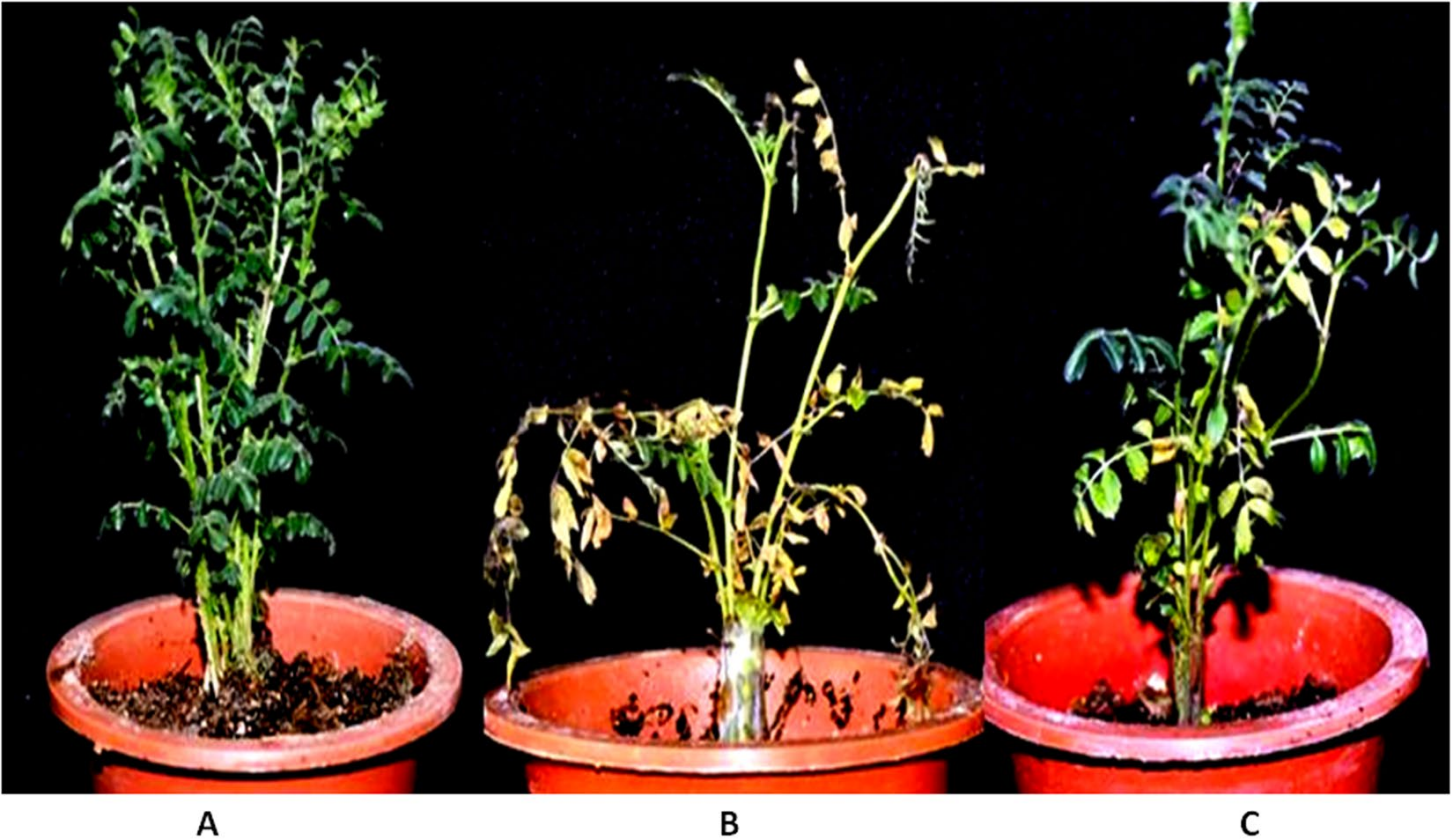
Bioprotection role of *P. indica* against Botrytis Grey Mould (BGM) disease. Chickpea plant infected with *B. cinerea* showing symptom of Botrytis grey mould (BGM) disease **(A)** Control plant not exposed to any fungus, **(B)** *B. cinerea*-infected chickpea plant is showing severe disease symptoms. **(C)** *P. indica* colonized chickpea plant infected with *B. cinerea*, showing decreased BGM disease symptoms.

We observed a significant enhancement of disease resistance in *P*. *indica-*colonized chickpea plants towards BGM disease caused by *B*. *cinerea*. We found plants colonized with the *P*. *indica* healthier and developed as compared to the *B*. *cinerea* infected plants. As shown in Fig. (8) plants infected with *B*. *cinerea* showed severe symptoms of BGM disease. Stems and leaves of the infected plants were found to be yellowish, curled and dried as compared to the plants inoculated with the *P*. *indica*.

## Discussion

Symbiotic relation is very important for plants as well as for the microorganism which helps them to cope up with the environmental adverse conditions. Microbes help host plant from both biotic and abiotic stresses. AMF association enhances growth of the host plant and provides tolerance against biotic and abiotic stresses^22,23^. *P*. *indica* mimics AMF and has strong association with a large number of medicinal and economically important plants. Since *P*. *indica* colonizes a variety of unrelated hosts and due to its beneficial nature, it is a putative bio-control agent and biofertilizer^19,24^.

We showed that colonization of the *P. indica* promotes the growth, biomass production and lowers the disease development caused by the pathogen *B. cinerea* in the economically important chickpea plant. Similar results have also been reported in case of *P. indica* colonized maize plant and later challenged by the *Fusarium*, thus support our data^19^. Similarly, it has been reported that *P. indica* protects *Arabidopsis* plant infected by soil-borne pathogen *Verticillium dahliae* by down regulation of the plant defence response^25^. Bio-protection role of *P. indica* against the leaf parasite *B. graminis* has also been shown by Waller and co-workers^3^. Authors have concluded that *P. indica* colonization with the barley plants helps in the development of resistance to *B. graminis* infection thus these findings support our data.

It has been shown that *P. indica* protects colonized maize and rice plants against the root pathogen *F. verticillioides* and parasitic fungus *B. graminis* respectively^3,19^. As we have observed that plants recovered their biomass when first infected with *B. cinerea* and later inoculated with *P. indica*. This suggests that *P. indica* acts as a bioprotective agent. We found that presence of the *B. cinerea* does not affect overall plant growth and development in the presence of *P. indica*. Our observations suggest the role of *P. indica* in bioprotection and disease resistance. In order to support our observation, with respect to whether inoculation with *P. indica* inhibits the colonization by *B. cinerea*, we have performed PCR analyses using *P. indica*- and *B. cinerea*-specific primers. We observed that in case of alternate as well as simultaneous interaction of both the fungi, an increased band intensity was noticed for *tef* gene of *P. indica* till day 30 and a decreased band intensity was observed in case of *cpr1* gene of *B. cinerea*. We conclude that gradual increase in the colonization by *P. indica*, results in the gradual decrease in the colonization of *B. cinerea* in the plants, which suggests that *P. indica* helps the plants to resist *B. cinerea* and helps the colonized plant to recover biomass as well as overall health.

Antibiosis assay demonstrates that there is no antibiotic/chemical secretion by *P. indica* to inhibit the growth of *B. cinerea*. This suggests that plant has developed the resistance towards the *B. cinerea* due to the colonization of the *P. indica*. Similar finding have also been reported by Kumar and co-workers^19^. Further authors have also found that inhibition in the growth of the pathogen *Fusarium* in the maize plant occurred due to the colonization of the *P. indica* and not by any antibiotic secretion. In another study by Deshmukh and Kogel^26^, it was reported that *P. indica* did not have direct inhibitory effect on the growth of the pathogenic fungus *Fusarium*. Recently, Rabiey and co-workers^27^ have also reported the similar findings during the interaction of *P. indica* with wheat plants infected with the *Fusarium*. Thus these studies support our hypothesis that resistance conferred to the chickpea plants by *P. indica* against pathogenic fungus *B. cinerea* is not due to any antibiotics or chemicals secreted by the *P. indica*.

To know whether root infestation by *P. indica* protects chickpea shoots from *B. cinerea* infections. Since *P. indica* does not infest shoot, protection against shoot pathogens would require a systemic response originate from the root, however, this phenomenon has not been reported for *P. indica* in case of chickpea plants. Therefore in the present study, we have also determined the antioxidant enzyme activity in root as well as in the case of shoot in chickpea plants colonized by the *P. indica* and infected with the *B. cinerea*. Inoculation of chickpea plants with *P. indica* strongly stimulates the activities of the antioxidative enzymes both in roots and shoots. In case of root and shoot we found 34 and 48 folds increase respectively in the GST activity during the colonization of the chickpea plants by *P. indica*. GST was found to be increased during the infection of Tobacco plants with *Colletotrichum orbiculare* ^28^. Similarly, Gullner and co-workers^29^ concluded that most likely role of GST is to reduce necrosis by detoxifying lipid hydroperoxides which are produced during the peroxidation of membrane. A consistent increase in GST level in our findings may also apply to the role of GST in limiting *B. cinerea* infection in chickpea plants, but this need warrant investigation.

It has been reported that CAT attenuate the elicitation of plant defence response by scavenging H_2_O_2_ and promotes intra-radical fungal growth^30^. In our study we found induction of CAT activities in case of chickpea plants infected with the *B. cinerea*. However, we observed a decrease in the CAT activities when plants first colonized by *P. indica* and later infected with the *B. cinerea* and *vice versa*. We suggest that less CAT activities results in the increase activity of H_2_O_2_ level which may lead to more production of the ROS that result in enhanced plant defence response against *B. cinerea*. In case of *F. culmorum* infected barley roots, a significant reduction in the activities of antioxidative enzymes like GR, DHAR, SOD, MDHAR and APX were observed^20^. Contrasting to this report, we found an increased activity of GR and SOD antioxidant enzymes in case of chickpea plants infected with *B. cinerea*. Similar results were also reported in case of maize plants infected with the *Fusarium*, thus support our data^19^. Elevated activities of antioxidant enzymes can be explained as this increased antioxidant activity minimizes the chances of oxidative burst and therefore *B. cinerea* might be protected from the oxidative defence system during infection.

It has been reported that pathogenic fungus *F. culmorum* infection with barley plants severely reduced growth. However, when plants pre-inoculated with *P. indica* and challenged with *Fusarium*, were found to be improved in the total biomass^19,20^. Interestingly, we have found the same pattern of reduced biomass in case of plant infected by *B. cinerea*. However, plants colonized with *P. indica* and later infected with *B. cinerea* recovered the loss in biomass caused due to *B. cinerea* infection as compared to control plant. This suggests *P. indica* colonizes roots of host plants and increases biomass and provide resistance against pathogenic fungus and, thus can be considered a bio-control agent. Under greenhouse conditions, it has been reported by Serfling and co-workers^31^ that due to the colonization of *P. indica* in winter wheat, symptom severity was found to be decreased significantly in case of infection by *B. graminis* f. sp. *tritici*, *Pseudocercosporella* and *Fusarium* and thus support our data.

Enhanced activity of SOD was found in case of plants colonized with the *P. indica* which may results in the accumulation of more H_2_O_2_, due to which less infection of *Fusarium* was observed in maize plants, therefore plants recover their biomass^19^. In the present study also, total activity of SOD was found to be increased in *P. indica* colonized plants as compared to the control plant, whereas when *P. indica* colonized plants were later infected with *B. cinerea*, the pathogen induced effects in SOD activity were attenuated as compared to plants infected with *B. cinerea* alone. This suggests, regulation of the SOD activity was much systemically repressed by *P. indica* in shoot and not repressed in case of roots in the presence of both fungi, however it needs further investigation.

It has been reported that *P. indica* and other fungal endophytes may enable plants to more efficiently scavenge ROS or prevent ROS production under stress conditions^20,32–35^. Our data suggest that antioxidant defence was maintained at a high level in plants colonized by the *P. indica* in response to *B. cinerea* infection. It is well-established that *B. cinerea* utilize production of ROS to accelerate cell death and facilitate subsequent infection^36,37^. We have observed a decreased antioxidant enzyme activity in the shoot in all the cases of alternate and simultaneous inoculations of *P. indica* and *B. cinerea* as compared to the plants only infected with the *B. cinerea*. We hypothesize that this decrease in the antioxidant enzyme activity would result in the induction of the oxidative stress exerted by ROS which may impart adverse effect in spread of *B. cinerea* infection. Therefore in all the cases we found an increase in the biomass of the *P. indica* inoculated plants as compared to the *B. cinerea* infected plants.

We have shown that colonization of *P. indica* with chickpea plants have strong growth promoting effects, systemic resistance to biotic stress and also results in the enhanced antioxidant capacity. Our study offers evidences that colonization of *P. indica* with chickpea plants might stimulate antioxidant enzymes which destroy ROS in plant cells to trigger subsequent defence reactions which in turn helps the chickpea plants to develop resistance against the infection of *B. cinerea*. As *P. indica* can be grown axenically, therefore, we suggest the use of *P. indica* as a bio-control agent in agriculture field to control pathogens and to overcome the use of pesticides and chemical fertilizers.

## Materials and Methods

### Plant and fungal culture and growth conditions

Chickpea seeds (*Cicer arietinum*, PUSA 1105) were surface-sterilized for 30 minutes in 2.5% sodium hypochlorite solution containing Tween-20. Seeds were finally washed in sterile distilled water few times and then imbibed in sterile distilled water overnight. A sterile filter paper was used in order to air-dry the seeds. Further, seeds were germinated by placing them (10 seeds) on Petri plate containing wet germination paper. For germination purpose, Petri plate contacting seeds was kept in the incubation at 23 ± 2 °C^38^. Seedlings were placed in pots (9 cm height and 10 cm diameter). *P. indica* (Varma, Kost, Rexer & Franken, 1997, European patent office, Muenchen, Germany. Patent No. 97121440.8-2105, Nov 1998) was a gift from Prof. Ajit Varma. *P. indica* was cultured in the laboratory routinely on solidified Aspergillus modified medium^39^ and were incubated for 7–10 days at 30 ± 2 °C. Growth of *P. indica* was studied in 250 ml culture flasks (containing KF media) with constant shaking at 100 rpm and 30 ± 2 °C in a metabolic shaker (Multitron Incubator Shaker, HT-Infors, Switzerland). *B. cinerea* was cultured on solidified potato dextrose agar (PDA) solid media or potato dextrose broth (1.5%) liquid media at 20–22 °C for 15–20 days. Surface sterilized pre-germinated chickpea seedlings were transferred to pots filled with a mixture of sterile sand and soil (3:1; garden soil and acid-washed riverbed sand). *P. indica* inoculation was performed as described previously^19^. Initially the plants were inoculated with *P. indica* through direct mixing of culture in sterile soil and by spraying *B. cinerea* grown culture suspension on the areal part of the plant. Ten millilitres of spore suspension (1 × 10^5^ conidia/mL) of *B. cinerea* was spray inoculated on chickpea plants. As control, one set of plants were grown without any fungus and mock treatment was given using autoclaved double distilled water. Chickpea plants were grown in a controlled environment in a green house at 23 ± 2 °C with a 12-hour light/dark photoperiod, a relative humidity of 60–70% and a light intensity of 1000 μmol/m^2^s. Twice in a week plants were given water and fertilized with Hoagland solution^40^. Plant samples were harvested at different time intervals after inoculation of the fungus (or mock treatment). These collected samples were carefully washed with running tap water, rinsed in deionised autoclaved water and weighed. Further, samples were frozen in liquid nitrogen for enzyme assay and root colonization analysis.

### Histochemical analysis

To study colonization, 10 randomly selected root samples were heated at 90 °C in 10% KOH for 15 min, followed by a treatment with 1 N HCl. They were stained with 0.05% trypan blue overnight or 60 °C for 1 h and destined in lactophenol^41,42^. Observation was done under the light microscope (Nikon Eclipse Ti). Percentage of colonization by the *P. indica* in the roots of plants was measured according to the method described previously^19,43^.

### Role of *P. indica* in bioprotection

All plants were initially grown for 10 days (day 0) and further, following sets were made: **(1)** chickpea plants grown for additional 30 days without any fungus (control); **(2)** plants were inoculated with *P. indica* at day 0 and then grown for 30 days; **(3)** plants were infected with *B. cinerea* at day 0 and grown for 30 days; **(4)** plants were first infected with *B. cinerea* at day 0 and then inoculated with *P. indica* at day 10 and grown for additional 20 days; **(5)** plants were first inoculated with *P. indica* at day 0 and later infected with *B. cinerea* at day 10 and grown for additional 20 days; **(6)** plants were simultaneously exposed to both fungi at day 0 and then grown for 30 days. Six pots each having 4 plantlets were used for each experiment.

For the analysis of root colonization or plant growth, the same experimental setup was used and the roots were harvested at different time points. The growth promoting effect of the two fungi was measured as fresh and dry weight. After the removal of plants from the soil, they were washed properly with water and surface moisture was evaporated on soft tissue paper. For the determination of the dry weight, plants were dried in an oven at 80 °C for 72 h.

The antibiosis activity of *P. indica* and *B. cinerea* was assayed by inoculating both fungi on the same plate. The appearance of margins of the growing fungi is the sign of antibiotic activity.

### PCR analyses

Bioprotection role of *P. indica* against the shoot pathogen fungus *B. cinerea* was checked by PCR as described previously^19^. For this purpose, following sets of plants were used **(1)** Chickpea plants inoculated with *P. indica* alone; **(2)** plants inoculated with *B. cinerea* alone; **(3)** Plants inoculated first with *P. indica* and later infected (at day 10) with *B. cinerea*; **(4)** Plants inoculated first with *B. cinerea* and later (at day 10) with *P. indica*; **(5)** Plants inoculated simultaneously with *P. indica* and *B*. *cinerea*. After 5, 15 and 30 days of inoculation, 10 plants were sampled from each set and examined for the presence of *P. indica* in plant roots tissues and *B*. *cinerea* within the shoot tissues. For PCR evaluation, total genomic DNA was isolated from the root and shoot tissues by the cetyltrimethylammonium bromide (CTAB) method as described previously^44^. PCR reactions were carried out in a final volume of 50 μl, containing 10 mM Tris-HCl (pH 8.3); 50 mM KCl; 1.5 mM MgCl_2_; 200 μM of dNTPs; 3 μM of each primer; 3 units of Phusion High-Fidelity DNA polymerase (Thermo fisher Scientific)) and 50 ng of DNA as template. To quantify the presence of *P*. *indica* and *B. cinerea* in chickpea plants, we analysed the expression of EF-1-alpha (*tef*) gene (AJ249912.1) of *P. indica*, the cpr1 (*cpr1*) gene (AJ609393.1) of *B*. *cinerea* and the elongation factor 1-alpha (*EF1-α*) (LOC101488243) of the chickpea plants by using the following primer pairs, for *P. indica*, Pitef-F (5′TCGTCGCTGTCAACAAGATG3′) and Pitef-R (5′GAGGGCTCGAGCATGTTGT3′) for *B. cinerea*, BCcpr1-F (5′GCTCGGCGCTACAAGAATTG3′) and BCcpr1-R(5′CAGCTTCACGTTCCTGGAGT3′) and for chickpea, CaEF1-α-F (5′TCACCATGGTTGCTGCTGAA3′) and CaEF1-α-R (5′ATATGACCACCGCCGATCAA3′). PCR reactions were performed at the following reaction conditions: denaturation at 95 °C for 2 min, one cycle; for 35 cycles, denaturation at 95 °C for 30 s, annealing at 58 °C, 59 °C and 58 °C for 30 s for *tef*, *cpr1* and the *EF1-α* gene, respectively; and extension at 72 °C for 30 s.

### Antioxidant enzyme activities in chickpea plants

Antioxidant activities were checked in presence or absence of the parasitic fungus *B. cinerea* and *P. indica*. The roots and shoots were separately frozen in liquid nitrogen and homogenized with an ice-chilled mortar and liquid nitrogen in QB buffer without 1,4-dithiothreitol [for SOD, CAT and GST assays]. For the GR assay, 50 mg polyvinyl pyrrolidone per gram of tissue was added. The crude homogenates were centrifuged at 15000 *g* for 15 min at 4 °C and the supernatants were frozen at −20 °C. The protein concentrations were determined by the method of Bradford using bovine serum albumin as standard^45^.

The SOD activity was determined as described by Roth and Gilbert^46^. One millilitre of reaction mixture contained 50 mM sodium phosphate buffer (pH 7.8), 100 µM EDTA, an appropriate amount of the prepared extract and 10 mM of pyrogallol. The enzyme activity was calculated by measuring the absorbance change at 420 nm for 120 seconds against a blank sample without extract.

CAT activity was assayed by measuring H_2_O_2_ disappearance using the method described previously by Beers and Sizer^47^. One millilitre of the CAT assay reaction mixture contained 0.05 mM sodium phosphate, pH 7.0, an appropriate amount of prepared extract and 1 mM H_2_O_2_. The decrease in H_2_O_2_ concentration was followed at 240 nm and the activity was calculated using the extinction coefficient of 40 mM cm^−1^ for H_2_O_2_. The blank sample contained no plant extract.

GST activity was determined as per the method described by Habig and co-workers^48^. One millilitre of reaction mixture contained 0.1 M sodium phosphate pH 6.5, an appropriate amount of prepared extract, 2% 1-chloro-2,4-dinitrobenzene and 1 mM reduced glutathione. The enzyme activity was measured at 340 nm for 180 seconds against a blank without the plant extract.

GR activity was determined by the oxidation of NADPH at 340 nm at 25 °C with the extinction coefficient of 6.2 mM cm^−1^ as mentioned by Nordhoff and co-workers^49^. The reaction mixture was composed of 100 mM potassium phosphate, pH 7.8, 2 mM EDTA, 0.2 mM NADPH and 0.5 mM glutathione (oxidized form) and an appropriate volume of the prepared extract in a 1 ml volume. The reaction was initiated by the addition of NADPH.

### Statistical Analysis

Statistical calculations were performed using Microsoft office excel 2007. ANOVA and Tukey’s test was used to test the significance of the data. For this purpose, Graph Pad Prism version 6 was used.
